# Increased NOX1 and DUOX2 expression in the colonic mucosa of patients with chronic functional constipation

**DOI:** 10.1097/MD.0000000000030028

**Published:** 2022-08-12

**Authors:** Xiuqin Wei, Mei Xue, Chunbo Kang, Lei Gao, Mengqiao Zhang, Chao Ma, Wei Jia, Yufeng Zheng, Lei Cao, Pan Chen, Shujing Jiang, Fong-Fong Chu, Qiang Gao

**Affiliations:** a Department of Gastroenterology and Hepatology, Center of Gastrointestinal Rehabilitation, Beijing Rehabilitation Hospital, Capital Medical University, Beijing, China; b Center of Digestive Endoscopy, Department of Gastroenterology and Hepatology, The First Affiliated Hospital, Henan University of Science and Technology, Luoyang, China; c Department of Acute Medicine, Queen Elizabeth Hospital, London, United Kingdom; d Department of Cancer Genetics and Epigenetics, Beckman Research Institute of the City of Hope, Duarte, California, United States.

**Keywords:** chronic functional constipation, cytokine, DUOX2, NADPH oxidase, NOX1

## Abstract

To determine whether oxidative stress and inflammation are associated with constipation by examining the expression of the main producers of reactive oxygen species, nicotinamide adenine dinucleotide phosphate (NADPH) oxidases, and pro-inflammatory cytokines in the colon of patients with chronic functional constipation.

The colonic biopsies were collected from 32 patients with chronic functional constipation and 30 healthy subjects who underwent colonoscopy. Colonic mucosal histology was observed. Interleukin (IL)-1β, IL-6, IL-8 messenger RNA (mRNA), and 4 members of NADPH oxidase (NOX1, NOX2, DUOX2, and NOX4) protein and mRNA were assessed by immunohistochemistry, western blotting, and reverse transcription polymerase chain reaction.

The tissues from both patients and healthy subjects showed normal histological structure without increase of inflammatory cells. NOX1 protein and mRNA levels were significantly increased compared to controls (*P* < .05). DUOX2 protein, but not mRNA, was increased by 2-fold compared to controls (*P* < .05). The levels of NOX2 and NOX4 protein and mRNA demonstrated no significant difference between patients and control subjects. The levels of IL-1β and IL-6 mRNA were significantly higher in constipation patients (*P* < .05), while IL-8 mRNA level was no different between the 2 groups.

NADPH oxidase and pro-inflammatory cytokine might be involved in the pathogeneses of chronic functional constipation.

## 1. Introduction

Constipation is a common gastrointestinal disorder, which is characterized by infrequent stools, hardened stool, or difficult stool passage. The median prevalence of constipation is 16% in all adults, while in the elderly population (60–101 years old), the prevalence is 33.5%.^[1,2]^ Constipation may be idiopathic or associated with a number of diseases or medications. Idiopathic or primary constipation is identified as functional constipation, and chronic functional constipation is defined as symptoms for at least 3 months.^[3]^ Chronic functional constipation negatively impacts on the patient’s physical health and quality of life. The cause of functional constipation can be multifactorial; including genetic predisposition, low fiber consumption, low socioeconomic status, lack of colonic movement, hormonal imbalance, etc.^[2,4]^ The pathogenesis of chronic functional constipation has not been completely elucidated; however, previous studies showed it may be associated with a range of conditions such as the reduction of colonic intrinsic nerves and loss of interstitial cells of Cajal,^[5]^ intestinal myopathy, intestinal dysbiosis,^[6,7]^ alteration in neurotransmitter signaling,^[2,8]^ and inflammation.^[9,10]^

5-hydroxytryptamine (5-HT, also known as serotonin), an important monoamine neurotransmitter mostly found in the intestines, was suggested to be involved in the pathogenesis of constipation.^[11,12]^ We have previously shown that 5-HT induced the expression of nicotinamide adenine dinucleotide phosphate oxidases (NADPH oxidase, NOX) in mouse colon and human colon cells in vitro.^[13]^ NADPH oxidase is the main generator of reactive oxygen species, which plays an important role in antimicrobial host defense and oxidative stress in the colon.^[14]^ In humans, there are 7 isoforms of NADPH oxidase, including NOX 1 to 5, dual oxidase (DUOX) 1 and DUOX2.^[15]^ NOX1 and DUOX2 are mainly expressed in the epithelial cells of the gastrointestinal tract.^[16,17]^ NOX1 plays a key role in mucosal immunity, inflammation, maintaining homeostasis of gut microbes, and promoting colon mucosal wound repair in the epithelial restitution.^[18,19]^ Similar to NOX1, DUOX2, a hydrogen peroxide producer in the intestines, also defends against invading microbial pathogens and actively participates in the signaling pathways against inflammation and other diseases.^[20–22]^ NOX2 is highly expressed in the endosome of phagocytes and is critical for antimicrobial host defense,^[23]^ whereas NOX4 is widely expressed in various tissues and has multiple pathophysiological functions.^[24]^ Whether these enzymes are involved in chronic constipation has not yet been studied.

We have previously reported that 5-HT augmented dextran sodium sulphate-induced colitis via gene expression of inflammatory cytokines including interleukin (IL)-1β, IL-6, and IL-8.^[13,25]^ IL-1β and IL-6 are pro-inflammatory cytokines and play important roles in the pathophysiological process of inflammation.^[26,27]^ IL-8, also known as CXCL8, is a chemokine with multiple functions in a variety of physiological and pathological processes.^[28,29]^ Recently, it was found that the serum levels of cytokine TNF-α, IL-1, IL-6, and IL-12 were reportedly increased in geriatric and pediatric patients with chronic constipation.^[10,30]^ It was speculated that the intestinal dysbiosis in patients with chronic constipation may lead to the elevation of serum pro-inflammatory cytokines.^[30]^ Inflammation was proposed to play a role in the pathogenesis of constipation.^[31,32]^ Therefore, it is important to evaluate the colonic expressions of inflammatory cytokines to determine if they may be involved in the pathogenesis of chronic functional constipation.

In this study, we examined the messenger RNA (mRNA) levels of IL-1β, IL-6, IL-8, and both mRNA and protein levels of NADPH oxidase NOX1, NOX2, DUOX2, and NOX4 in the colonic mucosa of patients with chronic functional constipation compared with healthy controls. Our results suggest that elevation of cytokines and NADPH oxidases in the colon could contribute to the pathogeneses of constipation.

## 2. Material and Methods

### 2.1. Participants

This is a case-controlled study performed in the endoscopy centers of Beijing Rehabilitation Hospital, Capital Medical University and the First Affiliated Hospital of Henan University of Science and Technology. Patients with functional constipation were selected using the Rome IV criteria.^[33]^ We enrolled 39 individuals (17 males and 22 females) with functional constipation symptoms for at least 3 months into the study. Their age ranged from 24 to 74 years with a mean age of 53 ± 14 years (mean ± standard deviation, years). Exclusion criteria were patients with other chronic diseases beside constipation, including irritable bowel syndrome with constipation, inflammatory bowel disease, chronic systemic diseases, metabolic diseases, cancer, diseases of the nervous system, a history of antibiotics use within the last 3 months or a history of receiving calcium-channel blockers. Thirty-eight asymptomatic healthy subjects were enrolled into the control group (20 female and 18 male) during routine medical examination. The age of the control group ranged from 30 to 71 years with a mean age of 52 ± 14 years. Both groups had similar composition in terms of age and gender (*P* > .05).

### 2.2. Tissue procurement

The colonic biopsies were collected from participants who underwent colonic endoscopy. The samples for RNA extraction were submerged in RNAlater (cat. no. 76106; Qiagen, Hilden, Germany) overnight to prevent RNA degradation. They were stored at –80°C along with samples for Western blotting. For histological analysis, the biopsies were fixed in 10% formalin for 12 hours, embedded in paraffin wax for staining with hematoxylin & eosin (H&E, Sigma-Aldrich, St. Louis, MO) and immunohistochemistry (IHC) as described below. We conducted IHC staining on the tissue samples from 17 patients and 18 controls, polymerase chain reaction (PCR) and western blotting from 32 patients and 30 controls, respectively, because of tissue sample availabilities.

### 2.3. Histopathology examination

The 4-μm slices were cut from distal colon tissue samples and stained with H&E for histological examination. The morphological features of the stained biopsies were evaluated under a light microscope to observe tissue structure and to enumerate infiltrating inflammatory cells according to previous description.^[34]^

IHC staining for NADPH oxidases was conducted as described previously.^[13,35]^ Briefly, tissue samples were stained with primary NOX1, NOX2, NOX4, and DUOX2 antibodies at 4°C overnight. The primary antibodies included goat polyclonal anti-NOX1 antibody (cat. no. ab121009; 1:2000 dilution; Abcam, UK), rabbit polyclonal anti-NOX2 antibody (cat. no. GTX12024; 1:500 dilution; GeneTex, Inc.), rabbit monoclonal anti-NOX4 antibody (cat. no. ab133303; 1:2000 dilution; Abcam), and rabbit anti-DUOX2 Ab (raised against a KLH-conjugated 501–600 amino acids of human DUOX2; cat. no. bs-11432R; Bioss, Beijing, China) (1:500 dilution). A rabbit polyclonal anti-β-actin antibody (cat. no. ab8227; 1:1000 dilution; Abcam) was used to detect β-actin protein for sample normalization. These primary antibodies for IHC were also used for western blot.

The antigen-antibody complexes were detected with secondary antibodies at 37°C for 30 minutes and subsequently probed with horseradish peroxidase-conjugated streptavidin. A 3,3′-diaminobenzidine reagent kit was used for color development, after which samples were counterstained with hematoxylin. The IHC staining was used to determine the protein levels of NOX1, NOX2, NOX4, and DUOX2 via a semiquantitative method previously described.^[13]^ The semiquantitative scores were calculated from staining intensity (none, 0; weak, 1; moderate, 2; strong, 3) multiplied by the percentage of positively stained cells (≤5%, 0; 6%–25%, 1; 26%–50%, 2; 51%–75%, 3; and >75%, 4).

### 2.4. Quantitative real-time PCR assay

PCR assay for NADPH oxidases was done as described previously.^[34,35]^ Total RNA was extracted using the TRIzol Reagent solution (Invitrogen) according to the manufacturer’s instructions. Two micrograms of total RNA was used for cDNA synthesis using PrimeScript RT Reagent Kit with gDNA Eraser (Takara, Japan) in a 40 μL reaction system following these steps: 37°C for 15 minutes, 85°C for 5 seconds, and 4°C for 10 minutes. The primers were designed by using the Primer3.0^[36]^ and synthesized Invitrogen (Table 1). Real-time quantitative PCR was done with an ABI7500 Real-Time PCR system (Applied Biosystems, Foster city, CA). A 2-step method was used because of the 60°C annealing temperature. The reaction procedure began with 95°C for 30 seconds, followed by 40 cycles of 95°C denaturation for 5 seconds and 60°C elongation for 40 seconds. The relative quantity of target gene mRNA expression was determined using the comparative Ct (2^–ΔΔCt^) method and normalized to the quantity of 18srRNA.

**Table 1:** Reverse transcription-quantitative polymerase chain reaction primers.

Gene	Primer (5′-3′)		Product (bp)
	Forward	Reverse	
NOX 1	TGTAGTGGGAGTTTTCTTATGTGG	AATATCGGTGACAGCATTTGC	71
DUOX 2	GGATACCGTCCTTTCCTAGACC	AGACACCAGGGGGCACC	91
NOX 2	TGTGTTCAGCTATGAGGTGGTG	GCTTCAGATTGGTGGCGTT	116
NOX 4	GTTTTCTGTTGTGGACCCAAT	TTATTGTATTCAAATCTTGTCCCAT	92
IL-1β	AGTGGTGTTCTCCATGTCCTTTGTA	AGCTTGTTATTGATTTCTATCTTGT	213
IL-6	ATGAGGAGACTTGCCTGGTGAAAAT	TCTGGCTTGTTCCTCACTACT	104
IL-8	AAGACATACTCCAAACCTTTCCACC	TTCAAAAACTTCTCCACAACCCTCT	170
18srRNA	GTAACCCGTTGAACCCCATT	CCATCCAATCGGTAGTAGCG	151

### 2.5. Western blotting analysis

Western blotting analysis was conducted as described previously.^[13,35]^ Protein lysates were prepared from collected tissues in ristocetin-induced platelet agglutination lysis buffer on ice by homogenization with a grinder. Thirty micrograms of protein from each sample were denatured and resolved by 10% SDS-PAGE then transferred onto polyvinylidene difluoride membranes. The anti-human antibodies used to detect NOX1, DUOX2, NOX2, and NOX4 proteins (see IHC for antibody information). The expression level of the target protein was determined by incubating the membranes with horseradish peroxidase-conjugated anti-rabbit immunoglobulin G and enhanced chemiluminescence reagent. A rabbit monoclonal anti-glyceraldehyde-3-phosphate dehydrogenase antibody (cat. no. ab9485; 1:1000 dilution; Abcam) was used to normalize for the protein loading. ChemiDoc XRS (Bio-Rad, West Berkeley, CA) was used to capture the images. The intensity of images was quantified with ImageJ 1.48v program (National Institutes of Health, Bethesda, MD).

### 2.6. Statistical analysis

All statistical analysis was performed by using the SPSS 22.0 statistics software (SPSS Inc., Chicago, IL). Data were reported as means ± standard deviations. The normality of distribution of experimental data was analyzed by Kolmogorov-Smirnov test. Then, for the parametrically data of patients versus controls, the independent 2-sample Student *t* test was used. Mann-Wilcoxon signed-rank test was used for nonparametric data of IHC and Kruskal-Wallis test for the data from different age groups. Values were considered statistically significant at *P* < .05.

## 3. Results

### 3.1. Morphological analysis

**Figure 1.**
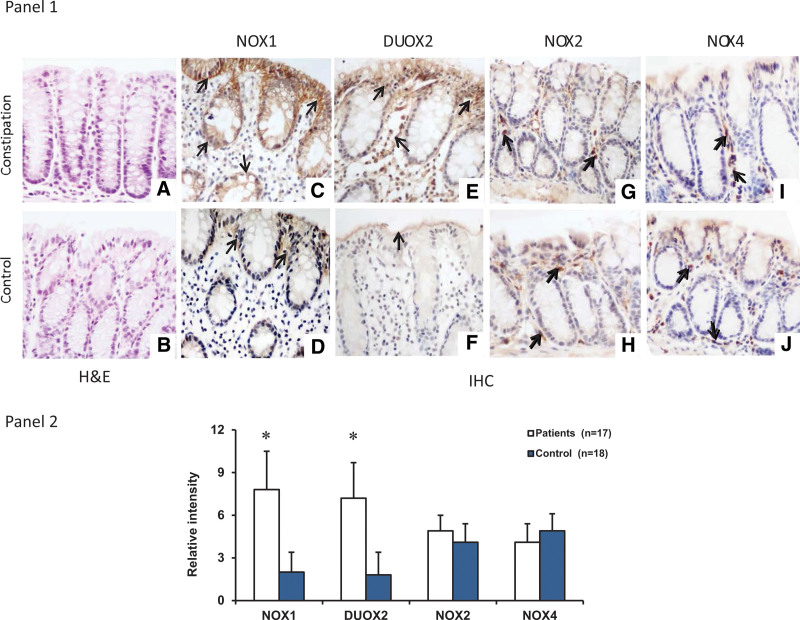
Histology and IHC staining of colonic biopsies. Sections from patients (A) with constipation and healthy subjects (B) showed normal tissue structure without infiltration of inflammatory cells (n = 15 for each group, H&E, 400×). Representative immunostainings for NOX1 (C and D), DUOX2 (E and F), NOX2 (G and H), and NOX4 (I and J). The expression of NOX1 was increased in the brush surface and cytoplasm of epithelial cell of crypts of colonic biopsies in constipation subjects (C) compared to control subjects (D). DUOX2 had a similar distribution pattern as NOX1, that is, the increased expression in the brush surface and cytoplasm of colon epithelial cells of constipation subjects (D), in control subjects, there was slight expression of DUOX2 in the brush surface of colon epithelial cells (E); NOX2 was mainly expressed in unidentified cells of the submucosa. There was no difference of NOX2 expression between constipation subjects (G) and control subjects (H); NOX4 had similar expression pattern to NOX2, that is, positive staining was seen in unidentified cells of the submucosa, there was no different the subjects in 2 groups (I and J). *P < .05, vs control group, n = 17 for constipation group, n = 18 for control group. DUOX = dual oxidase, H&E = hematoxylin & eosin, IHC = immunohistochemistry, NOX = nicotinamide adenine dinucleotide phosphate oxidase.

There were no apparent morphological differences in the colon biopsies from both patients with constipation and healthy subjects. These biopsies showed normal histological structure without increased levels of inflammatory cells (Fig. 1A and B). We did not observe neurons in the biopsies as the specimen size was too small.

### 3.2. The protein expression of NADPH oxidases in the colonic mucosa of patients with constipation

IHC staining and Western blot were used to determine the levels of 4 NADPH oxidases that were analyzed for mRNA expression. In IHC, NOX1 expression in the colon epithelial cells was increased in patients with chronic function constipation compared to control group (*P* < .05; Fig. 1C and D). NOX1 was expressed at the brush border membrane of colonic epithelial cells, as well as in the cell cytoplasm. DUOX2 protein expression was also elevated in patients with constipation compared with controls (*P* < .05; Fig. 1E and F). DUOX2 protein was mainly expressed in the colon epithelial cells with some found within the submucosa. NOX2 was primarily expressed in the infiltrating leukocytes within the submucosa and weakly expressed in the epithelium. The intensity of NOX2 expression was not different between patients and control subjects (*P* > .05; Fig. 1G and H). NOX4 had a similar distribution to NOX2, mainly in the infiltrating leukocytes. There was no difference in the levels of NOX4 expression between patients and control subjects (*P* > .05; Fig. 1I and J).

**Figure 2.**
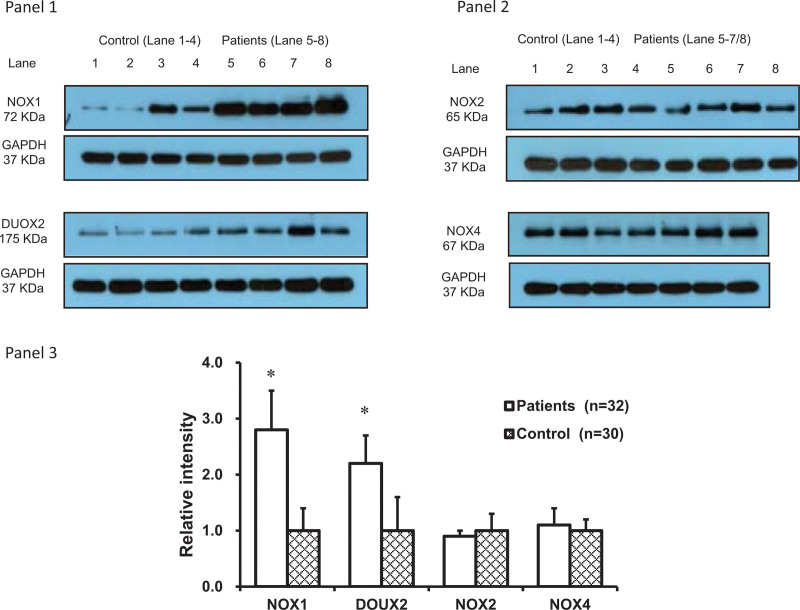
Western analysis of the protein levels of NADPH oxidases in colonic biopsies. NOX1, DUOX2, NOX2, and NOX4 protein levels were analyzed in the colonic mucosa of constipation patients and control subjects. Eight samples were analyzed for each protein from both groups except 7 samples were done for NOX4. *P < .05 compared to control subjects. DUOX = dual oxidase, GAPDH = glyceraldehyde-3-phosphate dehydrogenase, NADPH = nicotinamide adenine dinucleotide phosphate oxidase, NOX = nicotinamide adenine dinucleotide phosphate oxidase.

The data of Western blotting were shown in Figure 2, whereby NOX1 and DUOX2 protein levels were higher in the colonic mucosa of patients than the controls. NOX1 protein was increased by nearly 3-fold (*P* < .05), and DUOX2 protein was increased by 2-fold, compared to controls (*P* < .05). NOX2 and NOX4 protein levels were not different between the 2 groups. Furthermore, these 4 enzymes did not show statistically significant differences when comparison of patients genders and different ages (<50 years vs *>*50 years; both *P* > .05) (data not shown).

### 3.3. The mRNA expression of cytokines and NADPH oxidases in colonic mucosa of patients with constipation

**Figure 3.**
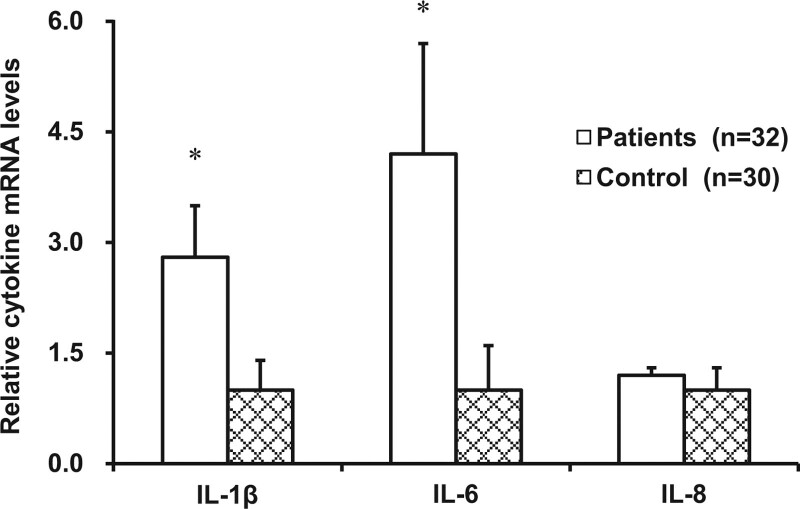
Pro-inflammatory cytokine gene levels. The levels of IL-1β, IL-6, and IL-8 mRNA were compared between 32 patients with constipation than 30 control subjects. *P < .05 compared to control subjects. IL = interleukin, mRNA = messenger RNA.

The levels of IL-1β and IL-6 mRNA were significantly higher in patients with constipation than subjects in the control group; increasing by nearly 3-fold and more than 4-fold, respectively (*P* < .05; Fig. 3). No difference was found in IL-8 mRNA levels between the 2 groups (*P* > .05; Fig. 3).

**Figure 4.**
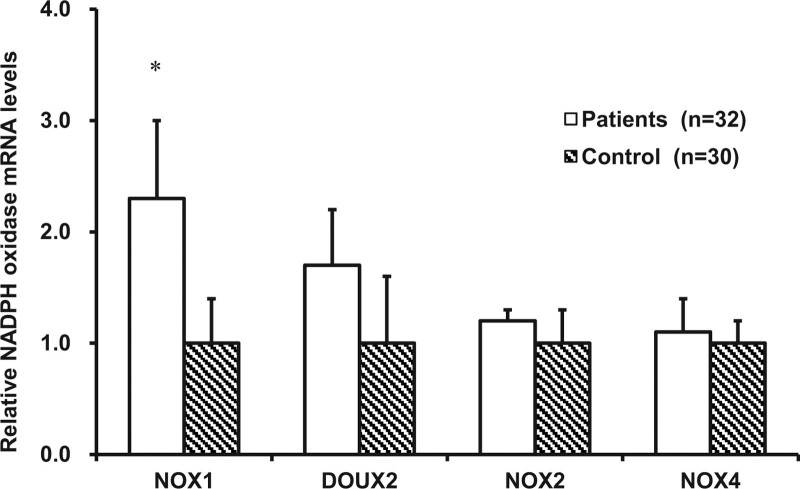
NADPH oxidases mRNA expression in colonic mucosa. The mRNA levels of NOX1, DUOX2, NOX2, and NOX4 in the colonic mucosa of constipation patients were compared with those of controls. *P < .05 compared to control subjects. DUOX = dual oxidase, mRNA = messenger RNA, NADPH = nicotinamide adenine dinucleotide phosphate oxidase, NOX = nicotinamide adenine dinucleotide phosphate oxidase.

We analyzed mRNA levels of 4 members of NADPH oxidases. NOX1 mRNA level in the colonic mucosa of patients with constipation was doubled compared to that in the controls (*P* < .05; Fig. 4). Although DUOX2 mRNA levels were higher in the patient group than the control, it did not reach statistical significance (*P* > .05; Fig. 4). The levels of NOX2 and NOX4 mRNA in the colonic mucosa of patients were similar to that of controls (*P* > .05; Fig. 4). By comparison of different genders and ages (<50 vs *>*50) of patients, there was no the statistical significance (both *P* > .05) at mRNA expression of these 4 enzymes (data not shown).

## 4. Discussion

This is the first study to report that NOX1 and DUOX2 protein expressions were increased in the colonic mucosal biopsy of patients with chronic functional constipation. It suggested that NADPH oxidases were involved in the pathogeneses of constipation. It was likely that the increased expression of these epithelial enzymes might be due to elevated 5-HT levels present in patients with chronic constipation.^[12,31]^ 5-HT was known to regulate gut motility and secretary functions.^[7,10]^ We have previously reported that 5-HT had a direct effect on colon epithelial cells resulting in increased expression of NOX1 and DUOX2.^[13]^ Individuals with chronic functional constipation had higher levels of 5-HT, and expression of 5-HT synthetic enzyme, tryptophan hydroxylase-1, in rectal biopsy samples than healthy volunteers as well as those having opiate-induced constipation.^[12]^ In this study, we did not find the expression of NOX1 or DUOX2 in colonic mucosa was associated with the gender and age of patients.

The mechanism of these enzymes in pathogeneses of constipation remained unclear. Since 5-HT was associated with the imbalance of intestinal microbiota, it is possible that dysbiosis was linked to the overexpression of NADPH oxidases in colonic mucosa of patients with constipation.^[11,37]^ It has been reported that gut microbiota uses MyD88 and the p38 signaling pathways to induce DUOX2 expression in colonic epithelia.^[38]^ DUOX2 modulates immune-mediated attack against invading microbial pathogens and actively participates in the signaling pathways against inflammation.^[20]^ The elevated expression of epithelial NOX1 and DUOX2 might interact with gut microbiota via their products to influence the physiological function of the colon. Redox signaling mediated by the gut microbiota via NOX1 in the intestines can influence a range of physiological functions, including the modulation of immune responses and enhancement of epithelial barrier function.^[39]^ Gut microbiota are critical for the development and activity of the intestinal immune system, including secretion of inflammatory mediator and enzyme, such as cytokine, chemokine, growth factor, and reactive oxygen species.^[40]^ Costa et al^[7]^ found that inflammatory potential of the diet was associated with the gut microbiota in individuals with functional constipation. It has reported that anti-inflammation or oxidative stress therapy alleviated experimental constipation,^[41,42]^ indicating that inflammation and oxidative stress was involved in the pathogenesis of constipation. We speculated that the intestine inflammation resulted in the interaction of gut 5-HT, microbiota and NADPH oxidases may be involved in the pathogenesis of constipation, as such, the mechanism of these factors in this process is worthy to be further explored.

We also found elevated mRNA levels of inflammatory cytokines IL-1β and IL-6 in the colonic mucosa of patients with constipation. This result suggested that inflammation was present in the colon, which supports that inflammation was a factor in the pathogeneses of chronic functional constipation.^[30,32]^ Since the histology analysis of colon biopsies did not show an apparent morphological change and IL-8 levels were not increased, the inflammation present must be very mild. The phagocyte NADPH oxidase NOX2 and NOX4, expressions were not elevated in the colon biopsies of patients with constipation, also supported this rationale. We believed that the elevated levels of IL-1β and IL-6 might be due to increased activity of resident cells that express these cytokines rather than an increased number of infiltrated inflammatory cells. Others have shown that inflammation was linked to constipation in both animal and clinical studies.^[10,31,32,43]^ In an animal study, Kim et al^[32]^ found that the treatment of *Asparagus cochinchinensis* to improve chronic constipation acted via the stimulation of anti-inflammatory responses, that is, the recovery of inflammatory cytokines, inflammatory mediators (iNOS), the number of mast cells, and mucin secretion. Wu et al^[44]^ suggested that in rat models, the improvement of constipation by Hesperidin was related to the increase of 5-HT receptor (R) 4 and intracellular-free calcium ions in the epithelium. Ren et al^[43]^ also found that enteromorpha and polysaccharides alleviated constipation-associated intestinal inflammation, decreased serum NO concentration, down-regulated vasoactive intestinal peptide receptor 1 expression and up-regulated 5-HTR4 expression in the distal colon, and altered intestinal microbiota. Concluding the results of the aforementioned studies, they indicated that 5-HT likely participate in the inflammatory mechanism of constipation.

In this study, we found only elevated NADPH oxidase and inflammatory cytokine IL-1β mRNA and IL-6 mRNA in the colon mucosa of patients with constipation. However, there were some limitations in this study. One limitation of the study is the lack of assay for the level of 5-HT expression in the colon mucosa. With data of 5-HT expression, we could have analyzed the correlation of these biological molecules with 5-HT. Second limitation is the lack of evaluation of the expression levels of these enzymes and cytokines after subjects underwent treatment of constipation to further confirm the role of these factors in constipation pathogenesis. Third point is that should more accurately evaluate inflammation of colonic tissue with more methods, such as periodic acid Schiff staining and identification of NADPH-positive stained cells.

In conclusion, our results suggest that inflammation might be involved in the pathogeneses of chronic constipation. The elevated NOX1 and DUOX2 expression may play a role in enhancing oxidative stress, which can contribute to constipation. The mechanism of these factors in the pathogenesis of constipation should be further explored in order to develop potential targets for the treatment of functional constipation.

## Author contributions

Conception and design: QG, XW, CK, LG; Patient recruitment and sample collection: XW, LG, MZ, MX, CM; Laboratory work: YZ, LC, PC; Literature search: QG, SJ, MX, F-FC; Provision of study material and subjects: YZ, LC, PC; article writing: QG, SJ, F-FC, CK; Funding acquisition: QG, CK, MX; All authors approved the final version.
